# Impact of Complying with a Procalcitonin-Guided Stopping Rule on the Duration of Antibiotic Therapy in Critically Ill Patients: A Real-Life Study

**DOI:** 10.3390/antibiotics14101012

**Published:** 2025-10-11

**Authors:** Edwige Péju, Auguste Dargent, Jean-Baptiste Roudaut, Sébastien Prin, Pascal Andreu, Audrey Large, Jean-Pierre Quenot, Pierre-Emmanuel Charles

**Affiliations:** 1 Service de Médecine Intensive-Réanimation, Hôpital F. Mitterrand, CHU Dijon Bourgogne, 14 Rue Paul Gaffarel, B.P. 77908, 21079 Dijon CEDEX, France; edwigepeju@hotmail.fr (E.P.); auguste.dargent@chu-lyon.fr (A.D.); jean-baptiste.roudaut@chu-dijon.fr (J.-B.R.); sebastien.prin@chu-dijon.fr (S.P.); pascal.andreu@chu-dijon.fr (P.A.); large.audrey@orange.fr (A.L.); jean-pierre.quenot@chu-dijon.fr (J.-P.Q.); 2 Lipness Team UMR 1231, LabExLipSTIC, Université Bourgogne Franche-Comté, 7 Boulevard Jeanne d’Arc, 21000 Dijon, France; 3 INSERM CIC 1432 Epidémiologie Clinique, Essai Clinique, CHU Dijon Bourgogne, 7 Boulevard Jeanne d’Arc, 21000 Dijon, France

**Keywords:** procalcitonin, antibiotic stewardship, sepsis, critically ill, intensive care unit

## Abstract

**Background:** Reducing critically ill patients’ exposure to antibiotics is mandatory. In randomized controlled trials, procalcitonin (PCT)-guided algorithms (i.e., antibiotic therapy [ABT] should be stopped whenever PCT is less than 0.5 µg/L or is below 80% of the peak value) reduced the duration of (ABT) more than compliance with the current guidelines. However, the interest of such stopping rules in daily practice remains debated. Thus, we carried out a real-life study addressing this issue. **Results:** During the study period, 112 patients with sepsis upon intensive care unit admittance were included. The median age was 66 years (56–79). Half of the patients presented with acute respiratory failure. Pneumonia was diagnosed in 78% of them, and 41% met septic shock criteria. The initial ABT was empirical in most cases, and appropriateness rate to the isolated bacteria reached 71%. A median number of four PCT measurements was achieved in both groups. The compliance rate with the PCT algorithm was 54%. The median duration of ABT was 5 (4–7) days if the PCT algorithm was followed, as compared to 7 (5–10) days otherwise (*p* < 0.001). This ABT stopping rule allowed a 2-day reduction in the treatment duration as compared with those recommended by the guidelines (*p* < 0.001). The only independent factor associated with shorter treatment duration was compliance with the PCT algorithm (OR = 0.74, 95% CI [0.62; 0.88]; *p* < 0.001). Regarding safety, no difference in outcome was found between the two groups. **Conclusions:** Complying with one PCT-based stopping rule is associated with a significant reduction in the duration of ABT in septic critically ill patients, without apparent impact on patient outcomes.

## 1. Background

Antibiotics are probably overused in hospitalized patients, especially in the intensive care unit (ICU) setting, thereby promoting the emergence of multidrug-resistant (MDR) pathogens and leading in turn to excess of mortality [1,2,3]. Reducing antibiotic resistance selection pressure is therefore mandatory and could rely, at least in part, on reduced treatment duration, as suggested by recent guidelines [4]. Besides this need, preventing the adverse effects of some antibacterial compounds including the beta-lactams could be critical regarding a patient’s outcome. Thus, it has been reported that cefepime neurological toxicity could be obvious as soon as the 4th day of therapy [5]. In addition, any supplementary single day of antibiotic treatment provides a 10% increase in the risk of serious adverse effects [6]. As a result, efforts should still be made to optimize antibiotic duration in critically ill patients.

In addition, reliably and safely assessing one single septic ICU patient’s response to antibiotic therapy (ABT) remains a matter of concern. In fact, differences in host immune response, underlying diseases, as well as incoming superimposed infectious or non-infectious complications alter clinical and biological components from one patient to another. Therefore, tailoring ABT duration is needed but remains a challenging issue.

Thus, given its appealing diagnosis accuracy for the diagnosis of bacterial sepsis and its fast kinetic correlated with patient outcome, PCT-based stopping rules likely to guide ABT duration management in an ICU setting have been successfully developed [7,8,9]. Accordingly, in several randomized controlled trials (RCTs), such strategies have proven to be safe and likely to reduce significantly antibiotic exposure of critically ill patients [10,11,12]. However, it could be difficult to translate those findings into practice improvements in a real-life setting.

Therefore, we carried out a pragmatic prospective study to assess the impact and the safety of the implementation of a PCT-based stopping rule on the duration of ABT in septic patients admitted in our ICU.

## 2. Results

### 2.1. Study Population and Septic Episode Description

**Figure 1:**
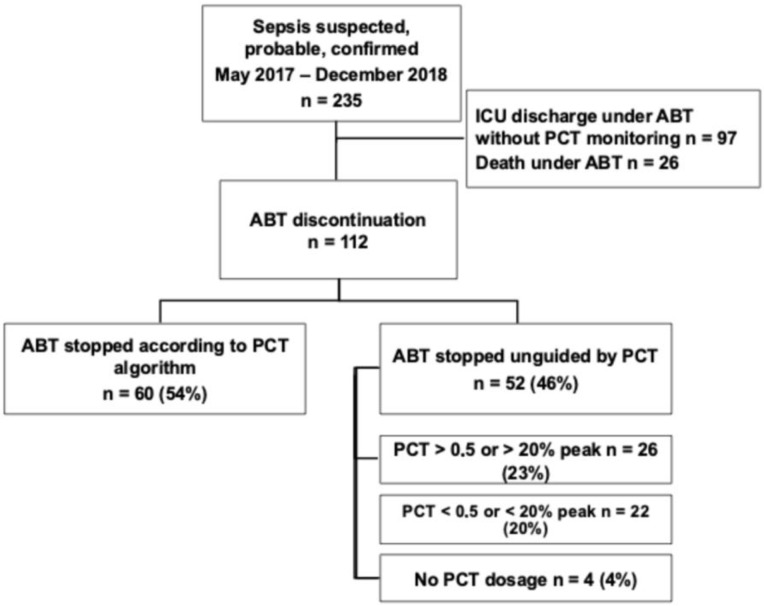
Flowchart of the study. ICU: intensive care unit; ABT: antibiotic therapy; PCT: procalcitonin.

From May 2017 to December 2018, 235 patients had a suspected or documented infection upon ICU admission. Among them, 26 patients (11%) died while ABT was still ongoing, and 97 patients (41%) left the ICU still under ABT without further PCT monitoring (Figure 1). We therefore included 112 patients.

The same number of PCT measurements was performed whenever patients were included in the PCT (4) [3,4,5] or in the standard of care group (4) [2,3,4,5] (*p* = 0.76) (Table 1). However, discontinuation of ABT was performed according to the PCT algorithm in 60 patients (54%), whereas 52 (46%) were treated according to the standard of care. Among those latter patients, 26 discontinuations (23%) were performed despite a less than 80% decrease in the PCT peak value, and 22 continuations (20%) despite a PCT value less than 0.5 μg/L or a more than 80% decrease in the peak value. Four patients (4%) did not have any PCT monitoring. Baseline characteristics of our population are detailed in Table 2. There was a male predominance (*n* = 79 [71%]), with a median age of 66 years old (56–79). Acute respiratory failure was the most common admission diagnosis. Most of the included patients were seriously ill since the SOFA score reached 7 (5–10) points and the SAPS 2 reached 48 (36–61) points. Moreover, in 46 (41%) cases, the infection was complicated with shock. Infection source was mainly pulmonary (*n* = 87 [78%]) and community-acquired (*n* = 51 [46%]). In more than half of the patients (*n* = 58 [52%]), the infection was documented, with bacteremia in 18% of cases. Gram-negative bacteria and especially enterobacteriacae were the most frequently involved species (*n* = 46 [41%]). Infection was polymicrobial in 29 patients (26%). Multidrug-resistant bacteria were isolated in 16 patients (14%), mainly in the setting of healthcare-associated infections. First-line ABT was empirical in almost all of the cases (*n* = 109 [97%]), and finally, appropriate in 71% of them.

### 2.2. Antibiotics Exposure and Compliance to the PCT-Guided Stopping Rule

**Figure 2:**
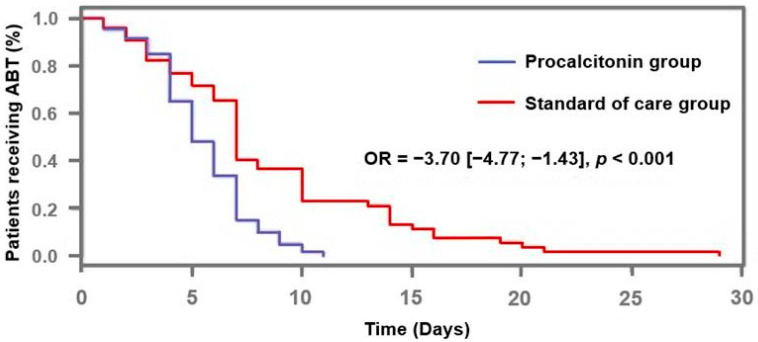
Proportion of patients receiving antibiotic therapy according to procalcitonin-algorithm. ABT: antibiotic therapy; OR: odds ratio.

ABT duration in the “PCT” group was significantly shorter than in the “standard of care” group (5.0 days [4.0–7.0] vs. 7 days [5.0–10.0], respectively; odds ratio [OR] = −3.70, 95% confidence interval [CI] [−4.77; −1.43], *p* < 0.001) (Figure 2). Moreover, in the “PCT” group, ABT duration was shortened by 2 days (−3.0–0.0) if compared to the guideline’s recommendations (Table 2), whereas the patients in the “standard of care” group were treated in accordance with them. The number of antibiotic-free days at day 28 was available in 99 patients, since 13 of them had to be excluded from this analysis because of missing data. It was found to be significantly greater in the patients from the PCT group than in others (7.0 [11.5] vs. 4.0 [8.0]; *p* = 0.019).

In an attempt to ascertain the link between ABT duration and compliance with the PCT algorithm, a multivariate analysis model was built. Interestingly, the duration of ABT was the only remaining variable independently associated with compliance to the PCT algorithm after adjustment for potential confounders such as clinical severity or source of infection (adjusted OR = 0.74; 95% CI [0.62; 0.88], *p* < 0.001) (Table 3).

### 2.3. Outcome

Resuming ABT within the 48 h following its interruption was equally frequent in both groups. Furthermore, there was no difference in terms of duration of mechanical ventilation, length of stay, ICU mortality, and 28-day mortality (Table 2).

## 3. Discussion

The main finding of our study is that complying with a PCT-guided ABT stopping rule in septic critically ill patients allows a significant decrease in antibiotic exposure, without apparent impact on patient outcome.

The first RCTs addressing those issues were carried out in patients admitted to emergency units with lower respiratory tract infections (LRTI) without any severity criteria. A significant reduction in ABT durations despite various PCT thresholds was shown, without compromising patient outcome [13,14,15]. Then, the studies focused on critically ill patients with suspected sepsis. The French PRORATA RCT included 621 patients with suspected bacterial infections and showed for the first time in an ICU setting that ABT discontinuation according to PCT value, and kinetic with time as well (PCT < 0.5 μg/L or a decrease of more than 80% of the peak), safely allowed a significant reduction by 2.7 days of the treatment duration [11]. These results were confirmed in an even larger RCT including 1575 patients, finding a 2-day ABT saving in the PCT group with the same algorithm, together with an unexpected decrease in mortality [12]. In a more specific population of patients with ventilator-associated pneumonia (VAP), the benefit of PCT in reducing mortality was also confirmed [16]. Other well-conducted studies were performed so far, with different algorithms (e.g., different PCT thresholds or decreasing rate) [9,13,17,18]. All of them found a significant reduction in ABT duration except the ProGUARD [19]. However, this was probably underpowered to detect the expected 2-day reduction of ABT in the PCT group. In addition, five meta-analyses conducted in critically ill septic patients confirm the benefit of using PCT to reduce the duration of ABT without impacting the outcome [20,21].

All those findings were, however, challenged with more recently published data, likely to demonstrate that PCT monitoring was finally useless for shortening ABT duration in patients with LRTI [22]. However, those latter studies included patients with mild severity infections, in whom very low baseline PCT values (e.g., lower than 0.25 µg/L in more than 90% of the patients) made unlikely the relevance of any biomarker-based stopping rule and hazardous any translation into the ICU setting, where greater ranges of values are usually encountered [9]. In addition, the 5-day mean treatment duration in the control group (i.e., fewer than recommended by the guidelines) suggests that the physicians did not really believe in the bacterial origin of the infection on a clinical basis. As a result, those latter findings support the fact that ABT could be safely shortened regardless of PCT values in patients with LRTI without any severity criteria, but any extrapolation to critically ill patients with sepsis from various origins remains hazardous. Lastly, physicians in charge were highly sensitized to ABT duration reduction prior to the beginning of the study. Regardless, two recently published RCTs that compared a PCT-guided protocol to the standard of care showed a significant reduction in ABT duration, along with a reduction in some ABT-related adverse effects [23,24].

It was also argued that RCTs assessing PCT-based algorithms were open-labeled studies, including highly selected patients, with various levels of compliance. That is why the so-called ProREAL study was conducted, showing that in real-life conditions PCT monitoring still allowed obvious reduction in ABT duration without deleterious effects on the outcome [14]. However, once more, only patients with LRTIs and low levels of severity were included in this study. We have previously published the results of a similar survey study conducted in our ICU, showing that in the subset of patients with VAP, compliance to the PCT algorithm could apparently safely reduce ABT duration [25]. Once more, the included patients, although seriously ill, were highly selected. Taken together, those findings emphasize the need for additional real-life studies in an ICU setting.

Thus, although obtained in a single center, the findings presented here are of potential interest. In fact, the patients’ characteristics as well as their outcomes were near those reported in previously published RCTs. Similarly and interestingly, in our study performed in “real-life” conditions, ABT duration in the “control” group (i.e., in which the PCT-stopping rule was not followed) was quite short (i.e., 7 days), in accordance with the latest guidelines but also as reported in the latest RCT that included critically ill patients, as was the 2-day reduction recorded if the PCT algorithm was followed [12].

Moreover, it is worth noting that despite an overall reduction in the duration of antibiotic therapy in our study, compliance to the PCT algorithm could also lead to longer lengths of treatment than the recommended ones in few cases. This finding emphasized the possible ability of PCT monitoring to match antibiotics exposure with one patient’s actual needs, especially in the most severe patients without a well-established infection source. This could be considered as a step towards personalized medicine.

**Figure 3:**
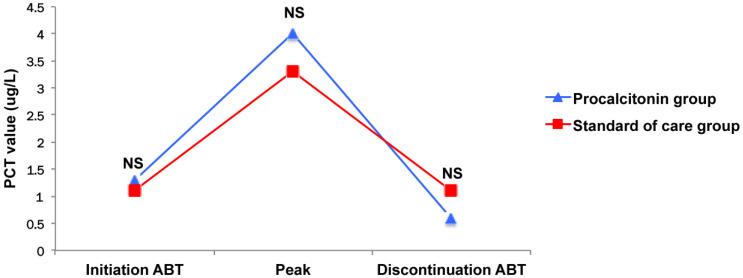
Kinetic of procalcitonin. ABT: antibiotic therapy; PCT: procalcitonin.

In the present study, despite the fact that the participating physicians were experienced, compliance with the algorithm reached 54%, the same as reported in the PRORATA study in which centers without any experience with the use of PCT were enrolled [11]. In contrast, higher rates (i.e., 68% overall and 82.5% in a subgroup of sensitized physicians) were reported in the PROREAL study [14]. Physicians are probably more reluctant to follow such a stopping rule in an ICU setting than in the general wards as well as the emergency department (ED), given the higher degree of clinical severity of the patients they have to deal with. Another explanation is the fact that obviously, the greater the PCT values are, the lower the compliance is. However, in our cohort, PCT values are similar regardless of the study group (Figure 3). We previously reported similar findings in VAP patients, suggesting that some physicians were maybe more influenced by PCT absolute values than its kinetic with time, despite the fact that the biomarker decrease magnitude was more strongly correlated to survival [8,19].

The observational design of our study is its main strength, as well as its weakness, although allowing us to consider it as a real-life study. In fact, physicians remained free to comply with the algorithm. This study has, however, several limitations. First, this was conducted in a single center. Second, the included patients were highly selected. This low rate of inclusion is mainly explained by the shortness of many ICU stays that did not allow completion of ABT in our unit, since PCT monitoring was unlikely in other wards. Third, the small sample size does not allow us to exclude any deleterious effect on the patients’ outcomes of the PCT algorithm implementation. As a result, our findings should be taken cautiously. Fourth, we should acknowledge that several years have elapsed since the study was conducted in the late 2010s. However, it is worth noting that guidelines regarding ABT duration in an ICU setting have not changed over this period.

Furthermore, it would be interesting to identify the individual practices of each prescriber, as it has been shown that long durations of ABT may be influenced by prescriber preferences rather than patient’s characteristics [26].

Finally, we did not perform a medico-economic study. This one could be interesting in order to reinforce the benefit of the follow-up of the algorithm of PCT, despite the expenses related to PCT measurement. Over the past years, numerous studies have shown that daily monitoring of PCT as a part of sepsis management promoted substantial cost savings in hospitals [27]. In addition, when this issue was adressed in one recent RCT, the cost-effectiveness of the implementation of a PCT-guided ABT stopping rule was demonstrated, even after a one-year follow-up [28].

## 4. Methods

### 4.1. Patients and Setting

We carried out a single-center observational prospective study in a 15-bed ICU. All patients over the age of 18 years admitted to our department with a suspected or documented infection meeting sepsis criteria, provided ABT was started upon admission or within the previous 24 h. Patients who died before ABT completion according to the PCT-stopping rule, as well as those discharged from the ICU without further monitoring of PCT, were excluded. Moreover, we excluded patients who needed a long duration of treatment according to the source of infection (e.g., endocarditis) or the involved pathogen (e.g., *Legionella*).

The institutional review board (Comité de Protection des Personnes Est I, Dijon) approved the protocol (2017-A01270-55) and considered it to constitute routine clinical practice. The need for informed consent was waived, but all patients or their relatives were given clear information about the study, and their non-opposition was obtained. 

### 4.2. Definitions

Sepsis was considered in patients with suspected or proven infection associated with any organ dysfunction (i.e., SOFA score reaching 2 points or more) caused by a dysregulated host response according to Sepsis-3 definitions [29,30]. Septic shock was defined as sepsis plus hypotension requiring vasopressor support despite adequate fluid filling, together with elevated blood lactate level beyond 2 mmol/L [30]. Multidrug resistant bacteria are defined according to international recommendations [31].

PCT assay was performed routinely with an immunofluorescent assay, according to manufacturer instructions (Range: 0.02–5000 µg/L; Thermo Scientific Brahms Kryptor^®^ analyser, Hennigsdorf, Germany).

### 4.3. Data Collections

Patients’ characteristics were extracted from electronic charts. For each patient, we collected the following: demographic data, type of admission, associated co-morbidities, admission severity according to the SOFA and the SAPS 2 scoring systems calculation [32,33], description and source of the septic episode, and administrated empirical ABT. Collection of nominative data was approved by the national authority for the protection of privacy and personal data (i.e., Commission Nationale de l’Informatique et des Libertés). 

In addition, the number of PCT measurements performed throughout the treatment period was collected for each patient, along with PCT levels achieved at both initiation and discontinuation of ABT, as well as the peak value.

### 4.4. Management of PCT-Guided ABT

Although the management of ABT duration remained at the discretion of each physician in charge, one PCT-based stopping rule was implemented in order to reduce ABT exposure in our patients. This rule was based on the algorithm successfully applied in the above-mentioned pivotal randomized controlled clinical trials. It was extensively described within the welcome booklet provided to every prescriber and reminded through regular training sessions. Physicians were thus advised to measure PCT upon admission in each patient with suspected sepsis and to obtain a new sample 6 h later if the first value was lower than 0.5 µg/L despite a high clinical probability of bacterial infection. Thereafter, physicians had been taught that measurements were required every 24–48 h, in order to catch PCT peak value, and then to evaluate its kinetic with time. Essentially, it was recommended to stop ABT as soon as PCT was less than 0.5 µg/L and/or than 80% of the peak value. Patients were assigned to the “PCT” group if the rule was followed. Otherwise, patients were assigned to the “standard of care” group, meaning that ABT duration was deemed to be in accordance with the current French guidelines [4].

### 4.5. Clinical Endpoints

The primary endpoint was the duration of ABT. The secondary endpoints were the 28-day number of ABT-free days, in the ICU and in the hospital as well, together with safety criteria including both ICU and hospital length of stay (LOS), duration of mechanical ventilation (MV), ICU mortality, and 28-day mortality rates, as well as the resumption rate of antibiotics within the 48 h following their stopping.

### 4.6. Statistical Analysis

For univariate analysis, the continuous variables were expressed as median (interquartile intervals) and qualitative variables as numbers (percentages). They were compared with a Mann–Whitney U test and an exact Chi-2 test (Fisher’s exact test if less than 5 values), respectively. The survival analysis was performed through Kaplan–Meier curves construction, which were compared according to a Log-rank test. Then, we performed a multivariate analysis based on a logistic regression model, including variables for which the associated regression coefficient had *p* < 0.20 by univariate analysis, and then removed the variables if *p* > 0.05 was obtained by multivariate analysis.

Statistical significance was set at a *p* value of < 0.05, and STATA software (10.1) was used for all analyses (College Station, TX, USA).

## 5. Conclusions

Procalcitonin monitoring safely reduced ABT duration in septic critically ill patients in one real-life study to the same extent as demonstrated in previously published RCTs. Obviously, there is still room for improvement regarding compliance with the PCT algorithm in an ICU setting, and at discharge as well. Then, further reduction in the overall exposure to antibiotics could be expected.

## Figures and Tables

**Table 1:** Outcomes according to compliance with the procalcitonin-based algorithm.

	Procalcitonin Group (*n* = 60)	Standard of Care Group (*n* = 52)	*p*
**Number of PCT measurements**	4 (3–5)	4 (2–5)	0.76
**PCT value at initiation of ABT (ug/L)**	1.3 (0.2–4.8)	1.1 (0.5–4.1)	0.52
**Peak value of PCT (ug/L)**	4.0 (0.9–10.4)	3.3 (1.1–15.7)	0.23
**PCT value at discontinuation of ABT (ug/L)**	0.6 (0.2–1.2)	1.1 (0.5–2.7)	0.70
**Duration of antibiotic therapy (days)**	5 (4–7)	7 (5–10)	<0.001
**Duration of antibiotic therapy as compared to guidelines (days)**	−2 (−3; 0)	0 (−1; 3)	<0.001
**Resumption antibiotic therapy within 48 h**	3 (5)	4 (8)	0.70
**Duration of mechanical ventilation (days)**	4 (2–10)	2 (0–7)	0.67
**Length of stay (days)**	9 (7–15)	5 (3–15)	0.28
**ICU mortality**	10 (17)	7 (13)	0.63
**28-day mortality**	9 (15)	11 (21)	0.40

Variables are expressed as median (interquartile range) or number (percentage) as appropriate. PCT: procalcitonin; ABT: antibiotic therapy; ICU: Intensive Care Unit.

**Table 2:** Patients baseline characteristics and sepsis description.

	Procalcitonin Group (*n* = 60)	Standard of Care Group (*n* = 52)	*p*
**Cause of ICU admission**			
**Acute respiratory failure**	37 (62)	17 (33)	0.002
**Septic shock**	8 (13)	12 (23)	0.18
**Hemorrhagic shock**	2 (3)	4 (8)	0.41
**Cardiogenic shock**	0 (0)	1 (2)	0.46
**Obstructive shock**	0 (0)	1 (2)	0.46
**Cardiac arrest**	4 (7)	9 (17)	0.14
**Coma**	4 (7)	3 (6)	1.00
**Framing invasive procedure**	0 (0)	1 (2)	0.46
**Voluntary medical intoxication**	3 (5)	0 (0)	0.25
**Others**	2 (3)	5 (10)	0.25
**Patients characteristics**			
**Male gender**	42 (70)	37 (71)	0.89
**Age (years)**	64 (55–78)	70 (61–79)	0.19
**Chronic renal failure**	4 (7)	14 (27)	0.004
**Chronic heart failure**	6 (10)	6 (12)	0.79
**Chronic respiratory failure**	4 (7)	7 (13)	0.34
**Cirrhosis**	0 (0)	10 (19)	<0.001
**Immunosuppression**	9 (15)	15 (29)	0.07
**ICU admission SOFA score (points)**	7 (4–9)	7 (5–10)	0.55
**ICU discharge SOFA score (points)**	2 (1–3)	2 (0–3)	0.70
**ICU admission SAPS 2 (points)**	46 (36–61)	50 (36–62)	0.56
**Sepsis main characteristics**			
**Septic shock**	24 (40)	22 (42)	0.80
**Infection type**			
**Community-acquired**	22 (37)	29 (56)	0.04
**Hospital-acquired**	21 (35)	18 (35)	0.97
**Healthcare-associated**	3 (5)	5 (10)	0.47
**Infection site**			
**Lung**	52 (87)	35 (67)	0.01
**Digestive tract**	2 (3)	7 (13)	0.08
**Urinary tract**	3 (5)	2 (4)	1.00
**Skin and soft tissue**	2 (3)	5 (10)	0.25
**Bone**	0 (0)	0 (0)	/
**Others**	0 (0)	3 (6)	0.10
**Unknown**	1 (2)	0 (0)	1.00
**Bacteremia**	9 (15)	11 (2)	0.39
**Documentation**	36 (60)	26 (50)	0.06
**Gram-positive bacteria**	12 (20)	7 (15)	0.52
**Gram-negative bacteria**	24 (40)	19 (42)	0.80
**Multidrug-resistant bacteria**	8 (13)	8 (15)	0.76
**Initial antibiotic therapy**			
**Empirical**	59 (98)	50 (96)	0.48
**Amoxicillin, Amoxicillin-clavulanic acid**	13 (22)	12 (23)	0.86
**Cephalosporins**	21 (35)	15 (29)	0.49
**Piperacillin-Tazobactam**	21 (35)	17 (33)	0.80
**Carbapenems**	4 (7)	7 (13)	0.34
**Aminoglycosides**	4 (7)	3 (6)	1.00
**Appropriateness**	22 (61)	19 (86)	0.98

Variables are expressed as median (interquartile range) or number (percentage) as appropriate. ICU: Intensive Care Unit; SAPS 2: Simplified Acute Physiology Score 2; SOFA: Sequential Organ Failure Assessment.

**Table 3:** Determinants associated with adherence to the PCT algorithm (multivariate analysis).

	Odd Ratio	95% CI	*p*
**Age (years)**	0.99	0.06–1.02	0.37
**Cirrhosis**	1.95 × 10^−9^	0.00–0.00	0.99
**Immunosuppression**	0.38	0.13–1.17	0.92
**SOFA score on admission (points)**	1.07	0.92–1.24	0.37
**Source of infection**			
**Lung**	4264.8	0.00–0.00	0.99
**Digestive tract**	1.23	0.05–30.00	0.89
**Urinary tract**	16.20	0.52–507.74	0.11
**Skin and soft tissue**	3.43	0.13–94.51	0.47
**Duration of ABT (days)**	0.74	0.62–0.88	<0.001

Logistic regression analysis was performed. The following variables were included into the model: cirrhosis, Immunosuppression, SOFA score on admission, source of infection and duration of ABT. CI: confidence interval; ABT: antibiotic therapy; SOFA: Sequential Organ Failure Assessment.

## Data Availability

The data presented in this study are available on request from the corresponding author due to privacy.

## Abbreviations

ABT: antibiotics; PCT: procalcitonin; ICU: intensive care unit; RCT: randomized clinical trial; VAP: ventilator-acquired pneumonia; LRTI: lower respiratory tract infection; CI: confidence interval; OR: odds ratio; SAPS II: simplified acute physiology score II; SOFA: sequential organ failure assessment.
